# A novel class of oral, non-immunosuppressive, beta cell-targeting, TXNIP-inhibiting T1D drugs is emerging

**DOI:** 10.3389/fendo.2024.1476444

**Published:** 2024-10-04

**Authors:** Gu Jing, SeongHo Jo, Anath Shalev

**Affiliations:** Comprehensive Diabetes Center and Department of Medicine, Division of Endocrinology, Diabetes, and Metabolism, University of Alabama at Birmingham, Birmingham, AL, United States

**Keywords:** TXNIP, TIX100, verapamil, islets, diabetes, oral medication

## Abstract

Diabetes treatment options have improved dramatically over the last 100 years, however, close to 2 million individuals in the U.S. alone live with type 1 diabetes (T1D) and are still dependent on multiple daily insulin injections and/or continuous insulin infusion with a pump to stay alive and no oral medications are available. After decades of focusing on immunosuppressive/immunomodulatory approaches for T1D, it has now become apparent that at least after disease onset, this by itself may not be sufficient, and in order to be effective, therapies need to also address beta cell health. This Perspective article discusses the emergence of such a beta cell-targeting, novel class of oral T1D drugs targeting thioredoxin-interacting protein (TXNIP) and some very recent advances in this field that start to address this unmet medical need. It thereby focuses on repurposing of the antihypertensive drug, verapamil found to non-specifically inhibit TXNIP and on TIX100, a new chemical entity specifically developed as an oral anti-diabetic drug to inhibit TXNIP. Both have shown striking anti-diabetic effects in preclinical studies. Verapamil has also proven to be beneficial in adults and children with recent onset T1D, while TIX100 has just been cleared by the U.S. Food and Drug Administration (FDA) to proceed to clinical trials. Taken together, we propose that such non-immunosuppressive, adjunctive therapies to insulin, alone or in combination with immune modulatory approaches, are critical in order to achieve effective and durable disease-modifying treatments for T1D.

## Introduction

Since the discovery of insulin over 100 years ago, there have been a lot of advances in the treatment of diabetes. However, the overwhelming majority of novel medications is aimed at Type 2 Diabetes (T2D). In contrast, insulin has remained the main approved treatment for Type 1 Diabetes (T1D). While insulin therapy has come a long way and there have been a lot of advances in the formulation of insulin and the technology of its delivery, including automated (closed-loop) insulin delivery systems, people with T1D still depend on multiple daily insulin injection or insulin infusions and there is still a lack of effective pharmacological approaches for T1D. Also, for decades the focus has almost exclusively been on identifying immunosuppressive and/or immunomodulatory approaches and this has indeed led to the FDA approval of teplizumab, an infusion regimen of humanized anti-CD3 monoclonal antibodies to delay progression from stage 2 (≥2 auto-antibodies, no symptoms) to stage 3 T1D (≥2 auto-antibodies, with symptoms) (1, 2). However, accumulating evidence from islet biology reveals that beta cells are not just ‘victims’ and rather play an active part in their own destruction and the pathogenesis of T1D (3). Since beta cells need to produce insulin, their level of protein synthesis is very high and as such they are more prone to endoplasmic reticulum (ER) stress. In addition, their relative lack of anti-oxidative enzymes such as superoxide dismutase, makes them more susceptible to oxidative stress. Thus, various factors such as metabolic stress or viral infection can initiate beta cell dysfunction, senescence, and death. This in turn leads to the release and formation of signals (e.g., chemokines, antigens) that can stimulate immune cells and trigger an autoimmune response. In fact, it has been suggested that such beta cell signals may precede T1D associated insulitis (4) and elevations in blood glucose have been demonstrated prior to the appearance of T1D auto-antibodies (5). It is therefore not surprising that purely immunosuppressive approaches have failed to yield the expected success. This has resulted in a major paradigm shift over the last several years that now recognizes beta cell pathology as an important factor that contributes to the pathogenesis of T1D and that needs to be addressed therapeutically (3, 5, 6). However, doing so has, until a short time ago, also been hampered by the lack of known, actionable targets. Nonetheless, some existing, orally available compounds have been studied in the context of T1D including among others the neurotransmitter, gamma aminobutyric acid (GABA) and the antihypertensive drug, verapamil as recently reviewed (7). However, while GABA has also been shown to act outside of the central nervous system and to exert beta cell protective and regenerative effect in preclinical mouse studies (8), a well-designed, randomized, placebo controlled trial failed to reach its primary endpoint of maintained C-peptide or beta cell function in recent onset T1D (9). On the other hand, verapamil has proven highly promising, demonstrating strong anti-diabetic effects in preclinical models as well as improvements in remaining C-peptide in independent human phase 2 and phase 3 trials in adults (10, 11) and children (12) with recent onset T1D. This is consistent with the fact that verapamil has been shown to downregulate the expression of TXNIP (13) and TXNIP in turn has been demonstrated to represent a promising target to preserve beta cells in T1D (14). In addition, this has led to the development of a new chemical entity, TIX100 (aka SRI-37330) (15), now specifically targeting the TXNIP signaling pathway believed to confer the beneficial verapamil effects (10, 13). TIX100 has just received clearance from the United States Food and Drug Administration (FDA) to start clinical trials and this Perspective therefore focuses on the identification, rationale, development, distinct properties and future implications of this novel class of TXNIP-inhibiting T1D drugs.

## Target identification

**Figure 1:**
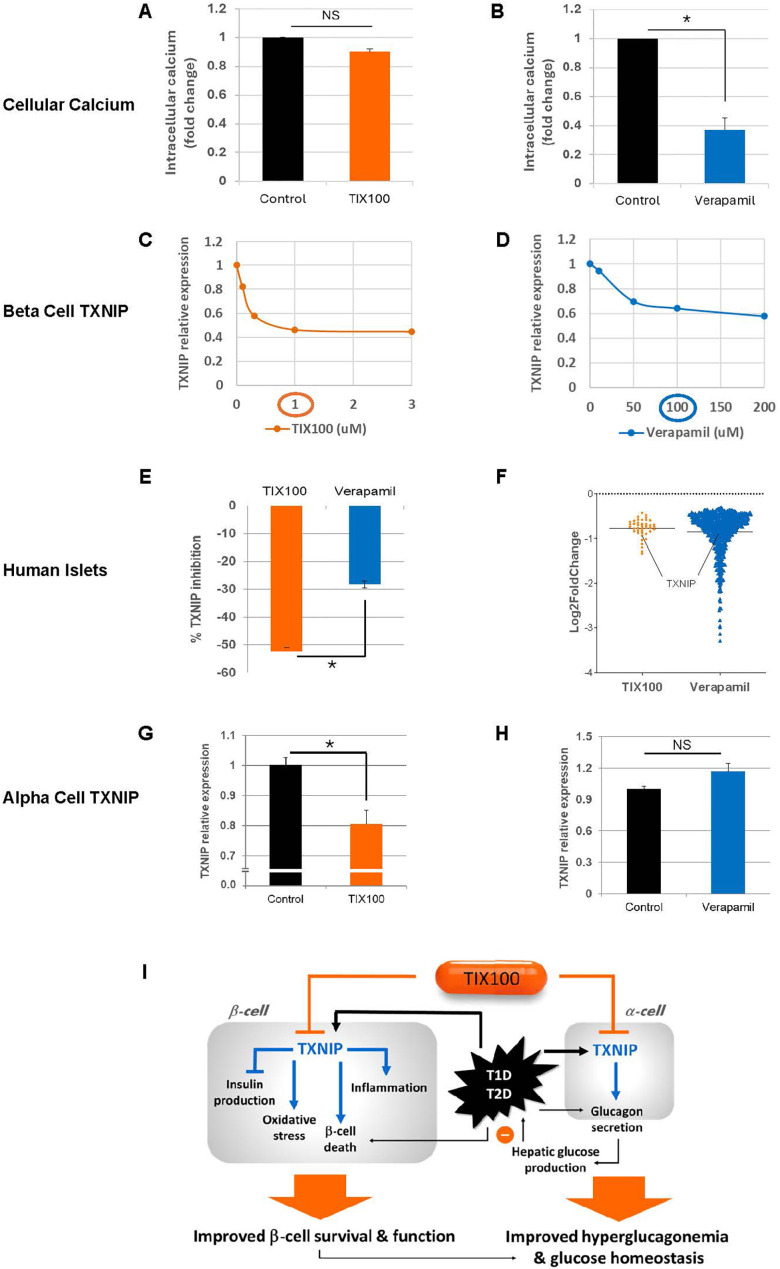
Additional insight into the effects of TIX100 and verapamil. Effects of **(A)** TIX100 (1µM) and **(B)** verapamil (100µM) on intracellular calcium of INS-1 cells as assessed by fluorometric calcium assay. Dose-response effects of **(C)** TIX100 and **(D)** verapamil on TXNIP expression as assessed by qPCR in INS-1 cells incubated for 24h at 11.1mM glucose. **(E)** Human islets were obtained from the Integrated Islet Distribution Program (IIDP) and were incubated for 24h at 25mM glucose and treated with TIX100 (1µM) or verapamil (100µM) and the % TXNIP inhibition was assessed by qPCR in the same 3 individual islets donors each serving as its own control. **(F)** Comparison of gene numbers found to be significantly downregulated (adjusted DESeq2 p-value < 0.05) by TIX100 (1µM) or verapamil (100µM) as assessed by RNA sequencing in the same 3 individual islets donors, each dot represents a gene and TXNIP is marked. **(G-H)** Alpha TC1-6 cells were incubated at 25 mM glucose and treated for 24h with TIX100 (1µM) or verapamil (100µM) prior to assessment of TXNIP by qPCR. Means ± SEM, n=3, *p<0.05, two-sided Student’s t-test, NS (not significant). **(I)** Schematic of the effects of TIX100 on alpha and beta cells and the implications for beta cell biology and glucose control.

TXNIP was originally identified as the top glucose-induced gene in a human islet gene expression profiling study (16). TXNIP is a 50kD cellular protein that binds and inhibits thioredoxin and thereby increases oxidative stress and impairs cell function and survival (17). However, the effects of TXNIP go beyond just inhibition of thioredoxin as it has also been demonstrated to play a major role in inflammasome activation especially in the context of ER stress (18, 19) and to modulate microRNAs involved in beta cell apoptosis and the regulation of insulin transcription (20, 21). In fact, TXNIP overexpression promotes beta cell apoptosis (14, 22, 23) and inhibits insulin production (20) (**Figure 1** **).** More recently, TXNIP (which is also expressed in non-beta cells) has been shown to promote diabetes-associated hyperglucagonemia and alpha cell glucagon secretion (24). TXNIP is well conserved across species and its expression is regulated primarily at the transcriptional level via an E-box motif in the TXNIP promoter (22). Importantly, TXNIP is not only induced by glucose *in vitro*, but pancreatic islet TXNIP expression is also elevated *in vivo* in various diabetes mouse models as well as in islets and beta cells of subjects with T1D and T2D (22, 23, 25). As such TXNIP is thought to contribute to a vicious cycle by further impairing islet function and in turn resulting in worsening of the hyperglycemia.

## Genetic proof-of-concept

The role of TXNIP as a detrimental factor in islet biology and contributor to the pathogenesis of diabetes has further been demonstrated by multiple groups and by genetic TXNIP deletions (14, 18, 19, 22). Whole body TXNIP deficiency and beta cell specific TXNIP deletion have been shown to protect mice against diabetes in models of T1D and T2D including streptozotocin (STZ)-induced beta cell destruction and genetic obesity and insulin resistance (14). Moreover, TXNIP was found to represent a critical link between glucose toxicity and beta cell death (23). TXNIP deletion also has beneficial effects in the context other tissues affected by diabetes complications including diabetic cardiomyopathy (26, 27), nephropathy (28, 29), retinopathy (30, 31), and neuropathy (32, 33) further supporting the notion of TXNIP representing an attractive target for systemic inhibition in the treatment of diabetes (17, 34).

## Pharmacological proof-of-concept

The elevated TXNIP expression found in human islets from individuals with T1D and T2D and the detrimental effects of increased TXNIP on beta cell survival and islet function provided a strong rationale for attempting to therapeutically inhibit islet TXNIP expression. In fact, the non–dihydropyridine L-type calcium channel blocker and approved antihypertensive drug, verapamil was found to non-specifically inhibit TXNIP expression (13). This effect is based on the verapamil-induced decrease in cellular calcium, also found with other calcium channel blockers or calcium chelators and is mediated by the inhibition of calcineurin signaling (13). Interestingly, verapamil was able to mimic the anti-diabetic effects of genetic TXNIP deletion observed in mouse models of T1D and T2D again using STZ-induced and obesity-induced diabetic mice treated with or without oral verapamil (13). Moreover, even when started after the onset of overt diabetes, verapamil was able to rescue the mice from diabetes due to STZ-induced beta cell destruction (13).

## Clinical supportive evidence

These pre-clinical findings have now been translated into humans as a phase 2 randomized, double-blind, placebo-controlled trial in adult subjects with recent onset T1D found that individuals receiving once daily, oral, slow-release verapamil to inhibit TXNIP for 1 year had improved beta cell function as assessed by mixed-meal stimulated C-peptide area under the curve (AUC), required less insulin, spent more time within the blood glucose target range, and had significantly fewer hypoglycemic events (10). Importantly, these beneficial effects seem to persist for at least 2 years with continuous medication (11). In addition, an independent phase 3 trial has now further validated the beneficial effects of verapamil in children with recent onset T1D (12). This provides supportive clinical evidence that targeting and inhibiting TXNIP has also anti-diabetic effects in humans with T1D and that (at least in the case of this target) the mouse models used were predictive of the translatability to humans. While verapamil was overall well tolerated in these smaller U.S. studies (10–12, 35), additional larger trials are still ongoing in Europe (NCT04545151) to prove its safety, tolerability and efficacy in this special population of subjects with T1D. In fact, as a calcium channel blocker, verapamil can cause arrythmias and potentially life-threatening atrioventricular heart blocks as well as hypotension limiting its use in some individuals. While no adverse cardiovascular events were observed in the adult studies (10, 11), the pediatric trial reported that in the verapamil group, 6% of participants with one or more nonserious adverse events of special interest, showed electrocardiogram abnormalities including prolonged PR interval, second-degree heart block, and first-degree heart block, and 2% developed hypotension as compared to 0% in the placebo group (12).

## New chemical entity for targeted therapy

Even though TXNIP has been validated as a promising therapeutic target for T1D, significant limitations are expected for the off-label use of verapamil to inhibit TXNIP for a T1D indication. Thus, a new chemical entity, specifically designed to inhibit glucose-induced TXNIP expression was developed using high throughput screening of 300,000 small molecules and extensive medicinal chemistry optimization resulting in TIX100, a substituted quinazoline sulfonamide (15). In contrast to verapamil, TIX100 does not function as an L-type calcium channel blocker (15) and as such does not pose a risk for the associated cardiovascular side effects. In addition, we have now confirmed that unlike verapamil, TIX100 does not alter cellular calcium concentrations (**Figures 1A, B**). TIX100 lowers TXNIP expression by specifically inhibiting the transcriptional activity from a conserved E-box motif of the TXNIP promoter (15). This inhibition is lost with mutation of just the first 7bp of this motif and thus seems to require the intact E-box repeat (15). Indeed, TIX100 was found to be highly effective in downregulating TXNIP expression in rodent and human islets (15).

Interestingly, our dose-response experiments now reveal that while TIX100 reaches its maximal TXNIP inhibitory effect at around 1µM, maximal TXNIP inhibition with verapamil occurs at around 100µM and a more than 100-fold lower concentration of TIX100 was sufficient to achieve comparable TXNIP inhibition (**Figures 1C, D**). With the molecular weight of both, TIX100 and verapamil being ~450 g/mol, this indicates a much higher potency of TIX100. Moreover, TIX100 is not only more potent, but also more effective than verapamil as suggested by its stronger maximal TXNIP inhibition observed in INS-1 cells (**Figures 1C, D**). We have now further confirmed this finding in human islets revealing a significantly bigger inhibitory effect in response to TIX100 as compared to verapamil in islets from the same donors (**Figure 1E**). Furthermore, RNA sequencing of human islets treated with/without TIX100 (15) or verapamil (11) revealed successful downregulation of TXNIP and its signaling pathway, however, while TXNIP ranked 7^th^ of a total of 42 downregulated genes in response to TIX100, it was number 192 of 619 decreased genes in response to verapamil (**Figure 1F**). Also, while in the case of TIX100 pathway analysis suggested regulation of energy, glucose and apoptosis in line with the known roles of TXNIP (15), verapamil seemed to affect a variety of pathways (11). This large number of off-target effects in the case of verapamil is consistent with its non-specific TXNIP inhibition and its role as a calcium channel blocker. It also highlights the contrast to the much higher specificity of the TIX100 effects on human islets.

As a small molecule, TIX100 is orally available and oral administration protected and even rescued mice from overt diabetes as shown in models of T1D and T2D including again STZ-induced and obesity-induced diabetes (15). In fact, TIX100 mimicked the anti-diabetic effects of genetic TXNIP deletion, whereas it had no additional beneficial effects in the absence of TXNIP, confirming its mode of action via TXNIP targeting (15).

Diabetes, including T1D and T2D, has long been recognized as a bi-hormonal disease characterized not only by absolute or relative insulin deficiency, but also by inappropriately high levels of its counter-regulatory hormone glucagon (36, 37). This hyperglucagonemia leads to excessive hepatic glucose production in the face of already elevated blood glucose levels and results in worsening of the hyperglycemia. In fact, inhibition of glucagon action has previously been shown to ameliorate glucose control in diabetes (38, 39). However, the applicability of such glucagon receptor antagonism approaches has been limited as they have also been reported to cause hepatic steatosis, liver enzyme abnormalities, alpha cell hyperplasia and hyperglucagonemia (39). In contrast, TIX100 decreases alpha cell glucagon secretion and serum glucagon levels and protects against hepatic steatosis without an increase in alpha cells or elevation in liver transaminases (15). Of note, TIX100 had no effect on glucagon secretion in the context of low glucose, which may help limit the hypoglycemic risk of TIX100. Indeed, even in the context of *in vivo* insulin-induced hypoglycemia, mice treated with TIX100 were able to defend their blood glucose levels equally well to untreated controls (15). This effect of TIX100 on glucagon secretion was also mediated by TXNIP inhibition (15) and mimicked by alpha cell-specific TXNIP deletion (24), but not observed in response to verapamil (10). In fact, we now have found that unlike TIX100, verapamil does not lower alpha cell TXNIP expression (**Figures 1G, H**), which helps explain why it does not have the glucagon-lowering effects. On the other hand, by controlling TXNIP in beta and alpha cells, TIX100 can improve beta cell health and function and also counteract hyperglucagonemia and excessive hepatic glucose production as summarized in our schematic (**Figure 1I**). These combined effects may explain the dramatic improvement in glucose homeostasis observed with TIX100 (15). Most recently, TIX100 completed all Investigational New Drug (IND) enabling safety and pharmacokinetic studies as well as chemistry, manufacturing, and control and has received clearance from the FDA to proceed to clinical trials. As such, there are now two TXNIP-inhibiting drugs available for clinical trials and we therefore provide a comparison of their currently known key features (**Table 1** **).**

**Table 1:** Comparison of key features of TIX100 and verapamil.

	TIX100	Verapamil	*References*
Controls beta cell TXNIP & improves beta cell health	✓✓	✓	(10–13, 15)
Controls alpha cell TXNIP & protects against hyperglucagonemia	✓	No	(15, 24)
Controls excessive hepatic glucose production	✓	No	(15)
Provides increased potency, effectiveness & specificity in TXNIP downregulation	✓	No	(11, 15), **Figure 1**
Maintains cellular calcium & avoids arrhythmia, heart block, or hypotension side effects	✓	No	(15), **Figure 1**
Reduces cellular calcium resulting in pleiotropic effects that might be beneficial in T1D	No	✓✓	(11), **Figure 1**

## Discussion

In summary, advances in the pharmacological treatment of T1D have been lagging behind those for T2D. Likely contributing factors include the predominant focus of industry on the larger market of T2D and, until recently, the over reliance of the field on technological advances and immunosuppressive approaches combined with the lack of good beta cell targets. Interestingly, the CLVer trial with its factorial design of participants receiving either intensive diabetes management with an automated insulin delivery system or standard diabetes care in addition to verapamil or placebo, nicely demonstrated that while advanced technology can yield optimal glucose control, this is not sufficient to impact beta cell pathology or delay disease progression (40) underlining the need for better pharmacological interventions. Indeed, the more recent realization that any disease-modifying T1D approach also needs to tackle beta cell pathology may lead to some novel breakthroughs. In this regard, targeting TXNIP inhibition seems to provide a promising approach. This is based on the fact that this approach targets an underlying disease pathology providing a strong rationale and that it has been validated in *in vitro* experiments, genetic mouse models, human islets studies and most importantly in adults and children with T1D (10–15, 18, 19, 22, 23, 41). Of note, based on the available preclinical data with verapamil and TIX100, TXNIP inhibitors are also expected to be useful in the treatment of T2D. In fact, several retrospective and a recent prospective clinical study with verapamil support this notion (42–44).

It is also worth noting that neither non-specific downregulation with verapamil, nor specific inhibition with TIX100 completely suppresses TXNIP expression (**Figures 1C, D**), yet effectively protected against diabetes in different preclinical models (13, 15). This is consistent with the stated therapeutic goal of just normalizing TXNIP to non-diabetic values and provides an additional safety margin (although even complete lack of TXNIP did not seem to cause any relevant detrimental effects in whole body TXNIP deficient mice (14).

While verapamil is immediately available for off-label use due to its FDA approval for hypertension and provides some control of beta cell TXNIP and improvement in beta cell health, it is associated with limitations due to its inherent risk for arrhythmias, heart blocks and hypotension and lacks other TIX100-associated benefits. Conversely, by reducing cellular calcium, verapamil has pleiotropic actions that go beyond TXNIP inhibition, and it remains to be seen whether these effects might provide additional benefits in the context of T1D or cause more side effects (**Table 1**). TIX100 has the advantage of higher specificity, potency, and effectiveness, and also improves hyperglucagonemia and excessive hepatic glucose production (**Table 1**), which obviously would also be beneficial in T2D. On the other hand, it still has to pass through the lengthy process of clinical trials to prove its safety, tolerability and efficacy in humans with T1D before becoming freely available in the clinic. Thus, verapamil provides a proof-of-principle for the translatability of the approach and may be helpful as an interim option as long as patients are carefully selected and monitored for any potential cardiovascular side effects. However, ultimately a more specific and targeted approach (such as with TIX100) could help avoid potential off-target effects while promoting the patient’s proper endogenous islet cell function. We propose that such an adjunctive oral therapy to insulin alone or in combination with immune modulatory approaches, holds high promise as an effective and durable disease-modifying treatment for T1D.

## Data Availability

The original contributions presented in the study are included in the article/supplementary material. Further inquiries can be directed to the corresponding author.
